# Two-tone distortion in reticular lamina vibration of the living cochlea

**DOI:** 10.1038/s42003-020-0762-2

**Published:** 2020-01-21

**Authors:** Tianying Ren, Wenxuan He

**Affiliations:** Oregon Hearing Research Center, Department of Otolaryngology, Oregon Health & Science University, Porrtland, OR 97239 USA

**Keywords:** Neurophysiology, Cochlea, Inner ear, Nanoscale biophysics, Hair cell

## Abstract

It has been demonstrated that isolated auditory sensory cells, outer hair cells, can generate distortion products at low frequencies. It remains unknown, however, whether or not motile outer hair cells are able to generate two-tone distortion at high frequencies in living cochleae under the mechanical loads caused by surounding tissues and fluids. By measuring sub-nanometer vibration directly from the apical ends of outer hair cells using a custom-built heterodyne low-coherence interferometer, here we show outer hair cell-generated two-tone distortion in reticular lamina motion in the living cochlea. Reticular-lamina distortion is significantly greater and occurs at a broader frequency range than that of the basilar membrane. Contrary to expectations, our results indicate that motile outer hair cells are capable of generating two-tone distortion in vivo not only at the locations tuned to primary tones but also at a broad region basal to these locations.

## Abstract (web summary)

Ren et al. used an in house heterodyne low-coherence interferometer to measure sub-nanometer vibrations, a proxy for distortion products, in living cochleae of gerbils. They were able to locate the generation source of the outer hair cell in the reticular lamina versus the basilar membrane in vivo.

## Introduction

**Fig. 1:**
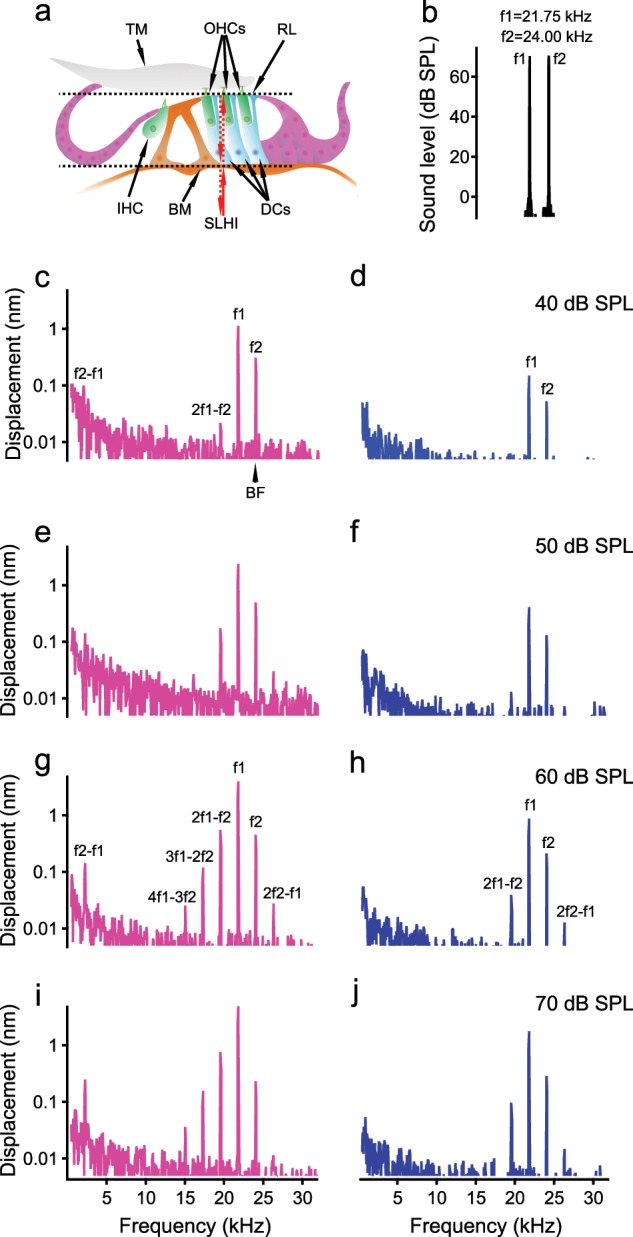
Diagram of a cross-section of the organ of Corti and magnitude spectra of stimulus, reticular lamina (RL), and basilar membrane (BM) vibrations. **a** Vibrations were measured from the apical ends of outer hair cells (OHCs) at the RL and from the BM using a scanning low-coherence heterodyne interferometer (SLHI) (magenta dotted lines). *IHC* inner hair cell, *DCs* Deiters’ cells, *TM* tectorial membrane. **b** The magnitude spectrum of a two-tone stimulus at 65 dB SPL (f1 = 21.75 kHz, f2 = 24.00 kHz). **c** The RL magnitude spectrum at 40 dB SPL shows that the RL vibrated not only at stimulus frequencies f1 and f2 but also at distortion frequencies 2f1–f2 and f2–f1. *BF* best frequency. **d** In contrast to **c** BM magnitude spectrum shows smaller f1 and f2 displacements and no distortion product. **e**–**j** As the sound level increased, response magnitudes at both primary and distortion product frequencies increased, and more distortion products emerged in both RL and BM vibrations.

When listening to two tones at frequencies f1 and f2 (f2 > f1), an individual with normal hearing perceives sounds not only at f1 and f2 but also at frequencies that do not exist in the stimulus, such as the two-tone distortion frequency 2f1–f2^1–3^. Such auditory illusion has an important role in sound perception because both speech and music include multiple frequencies^4,5^. Distortion products have been found in basilar membrane vibrations^6–10^, auditory nerve responses^11,12^, hair cell activities in vitro^13–15^, the intracochlear pressure^16^, and otoacoustic emissions (sounds emitted by the inner ear)^17^. However, the previous studies may not reliably reflect the cellular origin of distortion products for several reasons. First, the soft soma of Deiter’s cells may not effectively couple the outer hair cell motion to the stiff basilar membrane (DCs in Fig. 1a). Second, distortion products generated by isolated hair cells were observed only at low frequencies;^13–15^ and a recent in vivo study concluded that the frequency of outer hair cell motility is limited to < 3 kHz, far below the frequency the outer hair cells are expected to amplify^18^. Third, it is well documented that only sensitive living cochleae can amplify sounds^19–23^ and generate distortion product otoacoustic emissions^24–26^. The basilar membrane distortion^8,9,27,28^ and the reticular lamina nonlinearity^29–36^ vanish upon the death of the animals before the outer hair cells can be isolated. Thus, whether the outer hair cells can generate distortion products at high frequencies in living cochleae remains unknown. Here, we tested the hypothesis that the motile outer hair cells are capable of generating two-tone distortion in vivo by measuring sub-nanometer vibrations directly from apical ends of outer hair cells at the reticular lamina using a custom-built heterodyne low-coherence interferometer^29^. The current results show that reticular lamina distortion products are substantially larger and have broader spectra than those in the basilar membrane vibration. As the tops of outer hair cells are anchored to the reticular lamina, the current results indicate that motile outer hair cells are capable of generating two-tone distortion products at frequencies not only near the stimulus frequencies but also at a broad range of low frequencies. The current finding of the cellular origin of two-tone distortion in vivo is essential for understanding sound perception and for diagnosing auditory disorders by measuring distortion product otoacoustic emissions.

## Results

### Reticular lamina distortion products at different sound levels and f1 frequencies

As the apical ends of the force-producing cylindrical outer hair cells are part of the reticular lamina, we measured two-tone distortion products from the reticular lamina at the basal turn of living gerbil cochleae using a custom-built scanning low-coherence heterodyne interferometer (magenta dotted lines in Fig. 1a). When the object light of the interferometer was focused on the reticular lamina or basilar membrane^30^, the vibration was measured as a function of time at different sound pressure levels. A representative data set from 15 gerbils is presented in Fig. 1c–j. When two tones at frequencies 21.75 kHz (f1) and 24.00 kHz (f2) (Fig. 1b) were presented to the cochlea at 40-dB SPL equal f1 and f2 levels (0 dB SPL = 20 µPa), the reticular lamina vibrated not only at frequencies f1 and f2 but also at frequencies 2f1–f2 and f2–f1 (Fig. 1c). In contrast, the magnitude spectrum of the basilar membrane vibration shows smaller responses at stimulus frequencies f1 and f2 and no 2f1–f2 or f2–f1 above the noise level (Fig. 1d) at this sound level. As the sound level increased from 40 to 60 dB SPL, response magnitudes at both stimulus and distortion–product frequencies increased and more distortion products emerged in both reticular lamina and basilar membrane vibrations (Fig. 1c–h). At 60 dB SPL, the reticular lamina magnitude spectrum (Fig. 1g) shows as many as five distortion products (2f1–f2, 3f1–2f2, 4f1–3f2, 2f2–f1, and f2–f1), whereas the basilar membrane magnitude spectrum (Fig. 1h) shows only two of them (2f1–f2 and 2f2–f1). As the sound level increased further from 60 to 70 dB SPL (Fig. 1g–j), neither primary responses at f1 and f2 nor distortion product magnitude showed significant increase, indicating a compressive nonlinear growth. Moreover, reticular lamina and basilar membrane vibrations at f1 are significantly greater than those at f2, although the f1 and f2 sound level were equal and the vibrations were measured from the same f2 best-frequency location. Both the saturation and smaller f2 responses in Fig. 1c–j likely result from the nonlinearity and two-tone suppression. Thus, the data presented in Fig. 1c–j demonstrate two-tone distortion products in the reticular lamina vibration in the living gerbil cochlea, and show that they are significantly stronger than those on the basilar membrane.

**Fig. 2:**
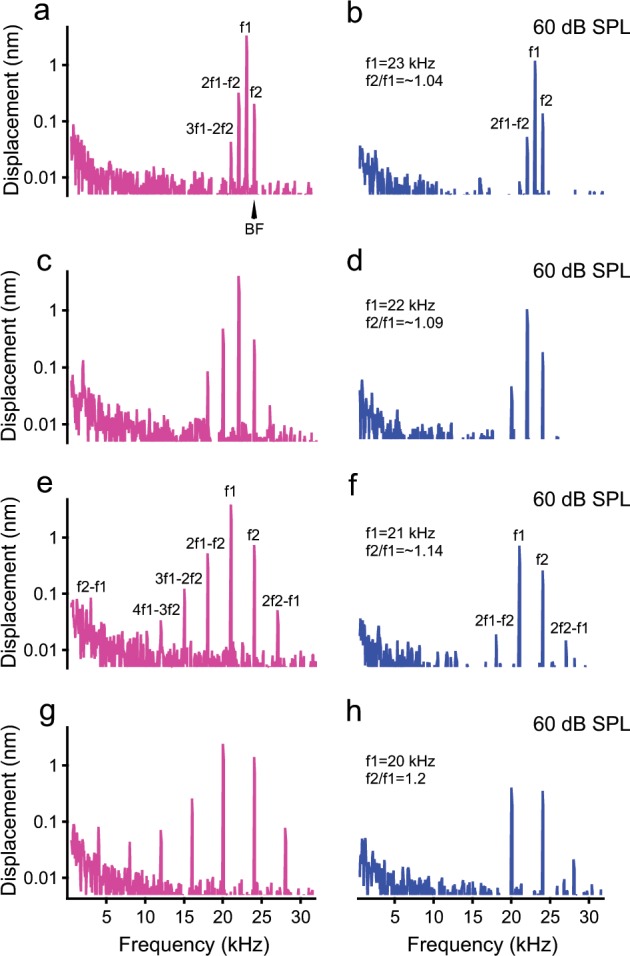
Magnitude spectra of reticular lamina (RL) and basilar membrane (BM) vibrations at different f1 frequencies. Data were collected at 60 dB SPL equal f1 and f2 levels, when f2 was kept constant at 24 kHz**. a**, **b** When f1 is close to f2 at 23 kHz, RL magnitude spectrum shows 2f1–f2 and 3f1–2f2 distortion products and only 2f1–f2 was observed in BM spectrum. **c**–**h** The RL and BM magnitude spectra when f1 was at 22 kHz **c**, **d** 21 kHz **e**, **f**, and 20 kHz **g**, **h**. As the f1 decreased from 23 to 20 kHz, and the f2/f1 ratio increased from ~ 1.04 to 1.2, the number of the RL distortion product increased from two (2f1–f2, and 3f1–2f2 in Fig. 2a) to five (2f1–f2, 3f1–2f2, 4f1–3f2, 2f2–f1, and f2–f1 in Fig. 2g). In comparison, the BM vibrations show fewer distortion products (Fig. 2b, d, f, and h).

Two-tone distortion products in the reticular lamina and basilar membrane vibrations measured at different f1 frequencies are shown in Fig. 2. The vibration was measured at 60-dB SPL f1 and f2 sound pressure level when f2 was kept constant at 24 kHz and f1 was varied. As the f1 frequency decreased from 23 to 20 kHz, and the f2/f1 ratio increased from ~ 1.04 to 1.2, the number of the reticular lamina distortion products increased from two (2f1–f2 and 3f1–2f2) in Fig. 2a to five (2f1–f2, 3f1–2f2, 4f1–3f2, 2f2–f1, and f2–f1) in Fig. 2g. Although the f2–f1 was not detectable in the reticular lamina vibration when f1 is close to f2 in Fig. 2a, it was obviously visible as the f2/f1 ratio increased in Fig. 2c, e, and g. The existence of the f2–f1 indicates that the cochlea is capable of detecting the temporal envelope of complex sounds mechanically^4,37^. In comparison, the basilar membrane vibrations show fewer distortion products and no significant f2–f1 (Fig. 2b, d, f, and h). The data in Fig. 2 indicate that two-tone distortion products depend on the f2/f1 ratio and this dependency is different between the reticular lamina and basilar membrane vibration.

### Relationship between distortion product and primary responses

**Fig. 3:**
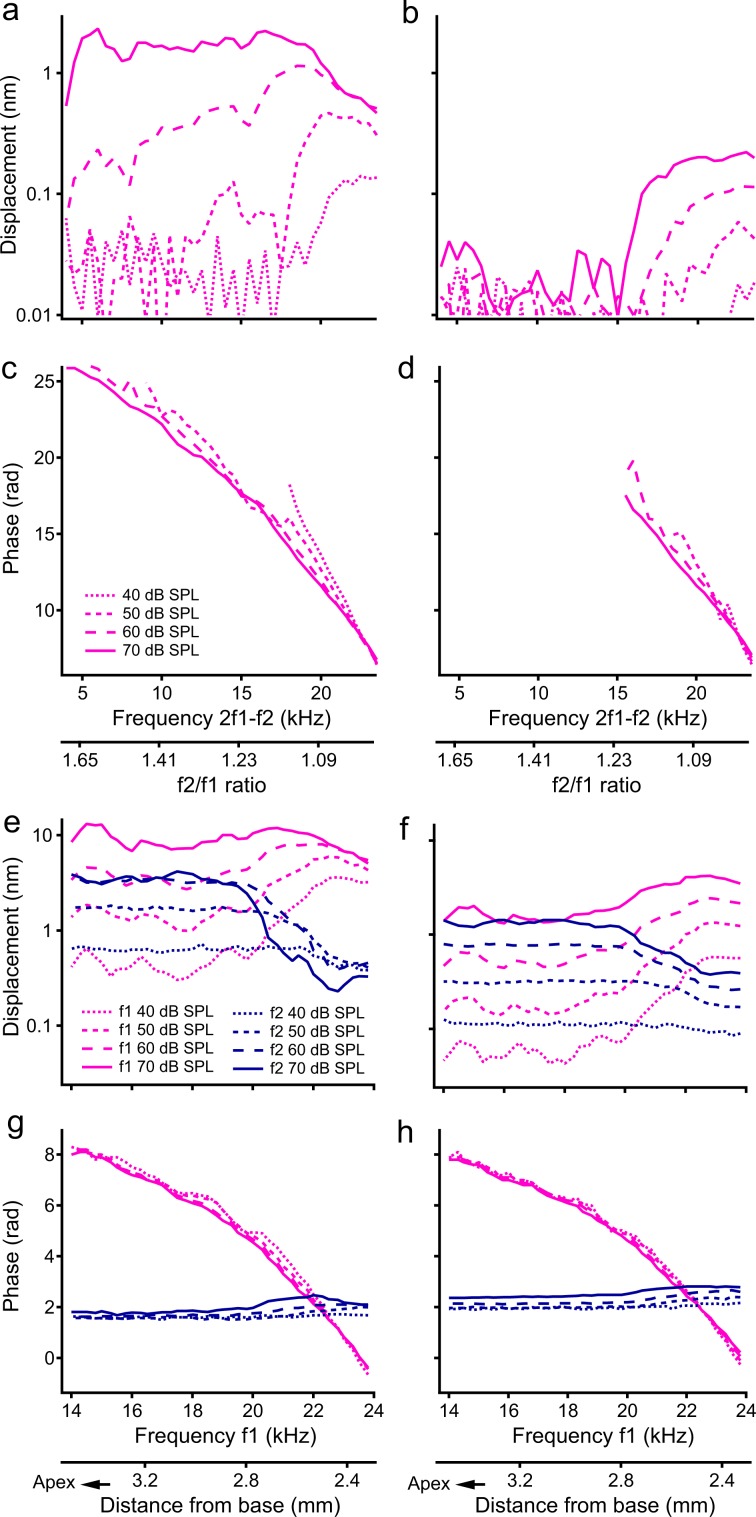
The magnitude and phase of the reticular lamina (RL) and basilar membrane (BM) vibrations at the distortion product frequency 2f1–f2 and stimulus frequencies f1 and f2. **a** The magnitudes of RL 2f1–f2 as a function of frequency at 40, 50, 60, and 70 dB SPL. As the sound level increased, the 2f1–f2 displacement increased nonlinearly at frequencies >20 kHz and its spectral bandwidth extended to low frequencies. **b** BM 2f1–f2 is much smaller than that of RL 2f1–f2 and has a narrow frequency bandwidth. **c** The RL 2f1–f2 phase deceases with frequency and the phase slope decreases with the sound level. **d** The BM phase data are similar to those of the RL. **e** Displacements of RL f1 and f2 as a function of f1 frequency at different sound levels. Although the f1 displacement increases with frequency and reaches maxima at ~23 kHz, the f2 magnitude decreases and reaches the minimum near this frequency. **f** BM f1 and f2 displacements are smaller and f2 responses show less saturation and suppression than those of the RL. **g** RL f1 phase decreases with f1 and f2 phase changes slightly. **h** BM f1 and f2 phase. The data were collected from a sensitive cochlea.

The magnitude and phase of the most commonly measured distortion product at 2f1–f2 were recorded from the reticular lamina and basilar membrane and presented as a function of the frequency (or the f2/f1 ratio) in Fig. 3a–d. At 40 dB SPL, the distortion product was observed in the reticular lamina vibration at frequencies >20 kHz with the f2/f1 ratio  <1.09 (magenta dotted line in Fig. 3a) but was hardly detected in the basilar membrane vibration at this sound pressure level (magenta dotted line in Fig. 3b). As the sound level increased from 40 to 70 dB SPL, both reticular lamina and basilar membrane distortion products increased in magnitude and expanded in frequency toward lower frequencies. For ~33-fold sound level increase from 40 to 70 dB SPL, the basilar membrane distortion product at frequencies near f2 increased 10-fold from 0.02 nm to 0.2 nm (or 20 dB), whereas the reticular lamina distortion product increased by only 3.7-fold from 0.13 nm to 0.48 nm (or 11 dB) and showed complete saturation at high sound levels. At 60 and 70 dB SPL, whereas the basilar membrane distortion product occurred at frequencies above 15 kHz, the reticular lamina distortion product expanded to the entire frequency range. Corresponding phases of the reticular lamina and basilar membrane distortion products are presented by magenta lines in Fig. 3c and d, respectively. Phase was measured in respect to distortion product phase at the stapes, which was calculated based on the measured f1 and f2 phase. At frequencies above 18 kHz and at a given sound pressure level, both the reticular lamina and basilar membrane phase decreased with frequency at a similar rate. The phase slope decreased slightly as the sound level increased.

To reveal the relationship between the distortion product and primary tone responses, the f1 and f2 magnitude and phase of the reticular lamina and basilar membrane vibrations were also measured as a function of the f1 frequency and presented in Fig. 3e–h. At 40 dB SPL, the f1 magnitude of the reticular lamina vibration increased with frequency and reached the maximum when f1 was close to f2, i.e., 24 kHz, the best frequency of the measured location (magenta dotted line in Fig. 3e). Although the f1 magnitude increased proportionally at frequencies below 20 kHz, it increased at a much slower rate and showed strong nonlinear compression at frequencies above 22 kHz (magenta lines in Fig. 3e). Despite the constant f2 frequency and sound level, the f2 magnitude decreased with f1 frequency and reached the minimum when f1 was close to f2 (blue lines in Fig. 3e). When the sound level was increased from 60 to 70 dB SPL, the f2 response showed no increase at f1 frequencies below 19 kHz, and a decrease at f1 frequencies above 20 kHz, which indicates a complete saturation and an increased suppression of f2 response (solid blue line in Fig. 3e).

Compared with the reticular lamina responses, the basilar membrane f1 responses are significantly smaller and show less compression at frequencies near the f2 (magenta lines in Fig. 3f). For sound pressure increase from 40 to 70 dB SPL, the basilar membrane f1 displacement increased by >6.2 times from 0.56 nm to 3.51 nm at the frequency near f2, whereas the reticular lamina f1 displacement increased by only ~1.5 times from 3.11 nm to 4.76 nm at the same frequency. The f1 tone-induced decrease in basilar membrane f2 response (blue lines in Fig. 3f) was smaller than that of the reticular lamina (blue lines in Fig. 3e). Reticular lamina f1 phase decreased with frequency (magenta lines in Fig. 3g) at a rate similar to that of basilar membrane phase response (magenta lines in Fig. 3h). Both the reticular lamina and basilar membrane f2 phase increased slightly as f1 frequencies approached f2, indicating a speed increase of the f2 traveling wave.

### Input and output functions of distortion products

**Fig. 4:**
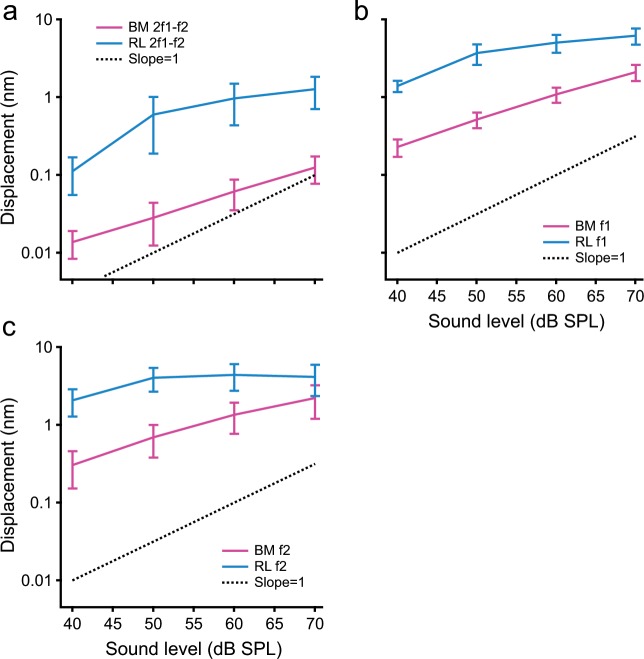
Input–output functions of the reticular lamina (RL) and basilar membrane (BM) distortion product and primary displacements. **a** Displacements of RL and BM distortion product 2f1–f2 as a function of the primary tone level. The dotted black line shows a proportional growth function. **b** Displacements of RL and BM f1 as a function of the primary tone level. **c** Displacements of RL and BM primaries f2 as a function of the stimulus level. Like primary f1 and f2, both RL and BM distortion products increased nonlinearly with the sound level. The data presented in this figure were collected from five animals and presented as mean and standard error.

Growth functions of the distortion product and primary tone responses are presented by 2f1–f2, f1, and f2 displacements as a function of the stimulus level in Fig. 4a–c. The data were collected from five sensitive cochleae with a constant f2/f1 ratio of 1.10. This ratio was used because both the reticular lamina and basilar membrane distortion products could be reliably measured under this condition. The magnitude of the reticular lamina distortion product is greater than that of the basilar membrane by ~10-fold, or 20 dB, at all four stimulus levels (*t* > 2.30, *p* < 0.05, *n* = 5). As the sound level increased, the reticular lamina distortion products increased and gradually saturated at high sound levels (turquoise line in Fig. 4a). For 30 dB sound pressure increase from 40 to 70 dB SPL, the reticular lamina distortion product increased by ~11 times from 0.07 to 0.80 nm, or ~21 dB, indicating ~9-dB compression. Similarly, the basilar membrane distortion product also increased nonlinearly with the sound pressure level by approximately nine times from ~0.01 to ~0.9 nm, or ~19 dB, revealing ~11-dB compression. The patterns of the 2f1–f2 growth functions of the reticular lamina and basilar membrane in Fig. 4a are similar to those of the f1 and f2 (Fig. 4b and c) despite magnitude differences.

## Discussion

The current results of heterodyne low-coherence interferometry demonstrate two-tone distortion in the reticular lamina vibration of sensitive living gerbil cochleae and its difference from the basilar membrane responses. The magnitude of reticular lamina distortion products is significantly greater than that of the basilar membrane and increases with the sound level, with nonlinear compression. In contrast to the limited frequency bandwidth of the basilar membrane distortion product (Fig. 3b), the reticular lamina distortion product extends to the entire measured frequency range (Fig. 3a). As the reticular lamina vibration was measured from the apical ends of outer hair cells, the present result indicates that outer hair cells are capable of generating two-tone distortion products under in vivo conditions, with mechanical loads from surrounding elastic supporting cells and tissues. In response to the two-tone stimulus, the motions of the reticular lamina^29–31,33^ and tectorial membrane^38–40^ result in a shearing motion in the subtectorial space, and deflect the hair bundles. The resultant bundle oscillation modulates conductance of mechanoelectrical transduction channels^41,42^, and hair cells consequently generate receptor potential^43,44^ not only at the f1 and f2 frequencies but also at distortion product frequencies owing to the inherent nonlinearity of the mechanoelectrical transduction^45–48^. These electrical distortion products are converted into cellular forces through electromechanical transduction^49^, hair bundle stiffness modulation^50^, and/or active bundle motion^42,51,52^. The resulting vibration at the apical ends of outer hair cells was detected by the interferometer in this study.

The nonlinear compressive growth of the reticular lamina and basilar membrane distortion products (Fig. 4a and b) indicates that the efficiency of distortion production decreases with the sound level^6^. This appears to be inconsistent with the fact that the compression of hair cell receptor potentials^53^ and single-tone responses of the basilar membrane^19^ and reticular lamina^29–34,54^ at high stimulus levels is stronger than that at low stimulus levels, which should increase efficiency of distortion production. However, the paradoxical decrease of distortion production efficiency likely results from the mutual suppression of primary responses f1 and f2 (Fig. 3e and f). As the distortion products result from f1 and f2, suppressed f1 and f2 responses consequently decrease the distortion product levels. Moreover, the magnitude of a single distortion product, such as 2f1–f2, may not accurately reflect the distortion production efficiency because the number of distortion products increases with sound level (Fig. 1c–j), indicating an energy redistribution of distortion products to more frequencies at higher sound levels.

Another important finding of this study is that the frequency bandwidth of distortion products of the reticular lamina is significantly broader than that of the basilar membrane (Fig. 3a and b). To illustrate the implication of this result, the f1 best-frequency location was calculated according to the frequency–location map in gerbil cochleae^55^ and shown below Fig. 3g and h as distance from the cochlear base. When f1 is close to f2 (24 kHz), or the f2/f1 is small, the f1 and f2 tone-induced vibrations largely overlapped, and distortion products were mainly generated near f2 best-frequency location (~ 2.26 mm from base)^7,9^. As f1 decreases, or the f2/f1 becomes larger, such as 1.6, there was no significant distortion product on the basilar membrane (Fig. 3b) but a robust distortion product at 6 kHz was recorded from the reticular lamina (Fig. 3a). Although the peak location of the f2 tone-induced vibrations was unchanged, f2 and f1 vibrations overlapped only at the basal part of the f1 vibration because the f1 vibration peak moved to a more apical location due to the smaller f1^56^, such as 3.52 mm from the base when f1 is ~ 14 kHz and the f2/f1 is ~ 1.65. Therefore, the broad frequency response of the reticular lamina distortion product indicates that two-tone distortion product otoacoustic emissions are generated not only at the best-frequency locations of primary tones, as commonly expected^24,57–61^, but also at a broad region basal to the f1 peak vibration location. The broad frequency response of the reticular lamina distortion product is also consistent with strong suppression of the reticular lamina f2 vibration over a broad f1 frequency range (Fig. 3e). The significant magnitude difference between the reticular lamina and basilar membrane distortion products at low frequencies (< 15 kHz in Fig. 3a) is probably owing to the loose mechanical coupling between outer hair cells and the basilar membrane and the stiffness difference inside the cochlear partition^62–64^. This interpretation is supported by our previous observation that outer hair cell-generated force in response to electrical stimulation cannot be effectively coupled to the basilar membrane at the cochlear location where the force is generated^65^. Instead, the outer hair cell-generated force initiates a forward basilar membrane traveling wave just as an external sound does^65^.

In comparison with previous studies^13–15^, the broad spectra of the reticular lamina distortion product indicates that outer hair cells can not only generate forces in vivo at low frequencies but also at high frequencies close to the tuned frequency of the measured location. The frequency limits of the outer hair cell electromotility caused by the membrane low-pass filter has been demonstrated in vitro for decades^66^. The discrepancy between the current experiment and previous in vitro studies likely results from changes in metabolic, chemical, electrical, and biomechanical status of hair cells under in vitro conditions. A recent in vivo study showed that the outer hair cell motility exhibits low-pass characteristics with corner frequencies ~3 kHz, which is >2.5-octaves below the tuned frequency of the observed cochlear location^18^. Although the outer hair cell and cochlear conditions in this experiment are comparable to those of the current study, a custom-designed complex stimulus with as many as 43 spectral components was used to evoke distortion products. Like two-tone-induced suppression shown in Fig. 3e, different components of the complex tone may have suppressed motile responses of outer hair cells to other components, which consequently reduces production of distortion products. This suppression-induced decrease of distortion products becomes stronger as the stimulus frequency increases and approaches the tuned frequency of the measured cochlear location. The current results, however, are consistent with commonly measured distortion product otoacoustic emissions^17,67^, and the broad spectra of the electrically evoked reticular lamina vibration in living cochleae^65^. These data suggest that, under the living conditions, force production of outer hair cells is not limited by the membrane time constant^68^.

In conclusion, heterodyne low-coherence interferometry demonstrates the two-tone distortion product in reticular lamina motion at a broad frequency range in the living cochlea. This result indicates outer hair cells are capable of generating two-tone distortion products under the mechanical loads of surrounding tissues not only at the tuned locations of primary tones but also at a broad region basal to these locations. This new finding is crucial for understanding the perception of complex sounds and for diagnosing auditory disorders in humans by measuring distortion product otoacoustic emissions.

## Methods

### Animals

Fifteen young healthy Mongolian gerbils of both sexes age 5–9 weeks (40–80 g) were used in this study. The animal use protocol was approved by the Oregon Health & Science University Institutional Animal Care and Use Committee. Data reported in this paper were collected from six sensitive cochleae. Results from the other animals were excluded because of an insensitive cochlea at high frequencies, poor signal-to-noise ratio, and/or incomplete data sets.

### Measurement of the cochlear partition vibrations

Experiments were conducted on a vibration isolation table inside an acoustically attenuated and electrically shielded double-wall booth. Under anesthesia induced by ketamine and xylazine (100 mg per kg and 10 mg per kg intramuscularly), a tracheotomy was performed and natural free breathing was maintained. Body temperature was kept constant at ~ 38 °C using a heating blanket that was feedback-controlled with a rectal temperature probe. The animal’s head was held firmly using a custom-built head holder mounted on a computer-controlled three-dimensional translational stage with rotation capability. A ventrolateral surgical approach was used to expose the left bulla and to transect the external ear canal. An acoustic probe connected to two speakers and a microphone was coupled to the remaining bony ear canal to form a closed sound field. Two tones at f1 and f2 frequencies of 20 and 24 kHz at 60 dB SPL (0 dB SPL = 20 µPa) were continuously presented to the ear canal, and the evoked distortion product otoacoustic emission at 2f1–f2 (16 kHz) was displayed on a dynamic signal analyzer (SR785, Stanford Research Systems, Sunnyvale, CA) and recorded through a digital lock-in amplifier (SR830 DSP, Stanford Research Systems, Sunnyvale, CA). Cochleae with < 5-dB decrease of the otoacoustic emission were considered sensitive. Hearing sensitivity was also confirmed by measuring the compound action potential of the auditory nerve using a round-window electrode^69^.

The anterior and lateral bony walls of the bulla were removed using a sharp blade to visualize the cochlea and the round window. About one-third of the round-window membrane was removed with a tungsten hook and the opened area was covered with a glass coverslip. Great care was taken to avoid bleeding by preserving blood vessels on the round-window membrane. When the basilar membrane was positioned approximately in the horizontal plane, a white light beam through a single-mode optical fiber was brought close to the lateral bony wall of scala vestibuli and scala media. The position and angle of the optical fiber were adjusted so that landmarks of the cochlear partition were visible through the microscope. Under direct visualization, low-coherence light from the object arm of the interferometer was focused on the center of the outer hair cell region through an infinity-corrected long working distance objective lens (Plan Apo × 20, NA 0.28, Mitutoyo, Japan). A custom-built scanning low-coherence heterodyne interferometer was used to measure vibrations inside the cochlear partition. This instrument has unprecedented sensitivity, temporal resolution, and spatial resolution for measuring micromechanical vibrations in living cochleae^29,65^. The wide dynamic range and low distortion make this interferometer particularly suitable for measuring low-level distortion product signals in reticular lamina vibration. After the basilar membrane and reticular lamina locations in the transverse direction were determined as described previously^30^, the object light beam of the interferometer was focused on those locations sequentially for vibration measurements. The locations of the basilar membrane and reticular lamina were indicated by the carrier signal level as a function of the transverse location and confirmed by the magnitude and phase of the cochlear partition vibration^30^.

### Signal generation and data acquisition

Two sinusoidal signals at frequencies f1 and f2 with durations of 20 ms and 1 ms onset and offset were synchronously generated by two digital-to-analog converters of a dynamic signal analyzer (PXI-4461, National Instruments, Austin, TX) at a sampling rate of 200,000 samples per second. These signals were used to drive two electrostatic speakers (EC1, Tucker-Davis Technologies, Alachua, FL) through two separate channels of a power amplifier. Two tones were delivered into the ear canal through separate coupling tubes. Sound level near the tympanic membrane was measured using a sensitive probe microphone and controlled by changing signal levels to the power amplifier. For determining the best frequency of the measured cochlear location, the magnitude and phase of reticular lamina and basilar membrane vibration were measured at different frequencies and sound levels. The best frequency was indicated by the frequency with the maximum magnitude of the basilar membrane response to 40-dB SPL tones. When f2 was kept constant at the best frequency, f1 was varied to evoke 2f1–f2 distortion products at different frequencies. The reticular lamina and basilar membrane vibrations were measured at 40, 50, 60, and 70 dB SPL of equal f1 and f2 tone levels. Higher sound levels, such as 80 dB SPL, were used only in a few animals. Distortion product at 2f1–f2 produced by the sound generation and delivering system was ~ 70 dB below f1 and f2 levels (Fig. 1b).

Output voltage from a displacement decoder (DD-500, Polytec Inc., Irvine, CA) of the interferometer was digitized by the analog-to-digital convertor of the same signal analyzer at the same sampling rate with the same time window as for signal generation. Acquired signals were averaged 20–50 times, depending on the signal-to-noise ratio. Spectra of the averaged signals were obtained through fast Fourier transform, and rms (root mean square) magnitude and phase of the displacement at primary tone and distortion product frequencies were determined.

To minimize animal heartbeat- and respiration-induced movement artifacts, the displacement signal from the decoder was reset to zero before data acquisition started. This made it possible to use a more-sensitive measurement range, such as 200 nm per V, for data acquisition. Owing to the heterogeneous nature of the tissues, particularly for the reticular lamina, animal movement often causes the backscattered light to change from one reflective surface to another. Resultant discontinuity of the 40-MHz carrier signal was detected by the decoder and converted into a 340 nm (0.5 wavelength) jump of the displacement signal. Data with such artifacts were identified and discarded during the averaging.

### Data analysis and statistical methods

Igor Pro (Version 7.0.8.1, WaveMetrics, Lake Oswego, OR) was used for processing and analyzing data. Different distortion products were shown by the magnitude spectra of the averaged displacement signal (Fig. 1c–j and Fig. 2a–h). The frequency responses of distortion products were displayed by the distortion product magnitude and phase as a function of the frequency (Fig. 3a–d). The magnitude and phase of the primary tones f1 and f2 were plotted as a function of f1 to show the mutual suppression of the two tones (Fig. 3e–h). Growth functions of distortion product and primary tones were presented by the displacements as a function of sound level (Fig. 4a–c). Magnitude difference between the reticular lamina and basilar membrane distortion products at a given sound level was determined by paired two-tailed *t* test and *p* < 0.05 was considered statistically significant. The grouped data were presented by mean and standard error calculated across the animals at given stimulus level, frequency, and cochlear condition.

### Reporting summary

Further information on research design is available in the Nature Research Reporting Summary linked to this article.

## Supplementary information

Supplementary Data 1

Supplementary Data 2

Supplementary Data 3

Supplementary Data 4

Description of Additional Supplementary Files

Reporting Summary

## Data Availability

Source data underlying plots in Figs. 1–4 are in Supplementary Data 1–4 and all other data (if any) are available from the corresponding author upon reasonable request.
