# Supplementary material for: Two-tone distortion in reticular lamina vibration of the living cochlea
Source: Commun Biol. 2020 Jan 21;3:35. doi: 10.1038/s42003-020-0762-2 (PMC6972885; doi:10.1038/s42003-020-0762-2)
Supplement: Supplementary file 5 — Description of Additional Supplementary Files [file 42003_2020_762_MOESM5_ESM.pdf]

Description of Additional Supplementary Files

File Name: **Supplementary Data 1**
Description: Source data underlying plots in Figure 1.

File Name: **Supplementary Data 2**
Description: Source data underlying plots in Figure 2.

File Name: **Supplementary Data 3**
Description: Source data underlying plots in Figure 3.

File Name: **Supplementary Data 4**
Description: Source data underlying plots in Figure 4.
